# Supplementary material for: Learning curve and surgical outcome of robotic assisted colorectal surgery with ERAS program
Source: Sci Rep. 2022 Nov 29;12:20566. doi: 10.1038/s41598-022-24665-w (PMC9709162; doi:10.1038/s41598-022-24665-w)
Supplement: Supplementary file 1 — Supplementary Information 1. [file 41598_2022_24665_MOESM1_ESM.zip › LSC Raw data-Ñ[▒K/070/H_ñw╝╨░O▒Kñσ.pdf]

正式摘要(電子簽章：000000186432682)

W83 / W83 ☐ 英文標題(注意:無英文姓名-請補病人基本檔!) ☐【讀取】憑證

臺中榮民總醫院

出院病歷摘要

轉入號： 轉出號：

[redacted]

(8)流水編號	
(9)入院日期	109/02/19 (2020) 直腸外科 W83 - 053
(10)轉科日期	
(11)出院日期	109/02/26 (2020) 住院天數計 7 日

診斷

- 入院 :Rectal tumor, s/p 5cm FAV s/p Per-anl excision on 2020/01/16
- 出院 :Rectal tumor, s/p 5cm FAV s/p Per-anl excision on 2020/01/16, s/p low anterior resection with colo-anal anastomosis laparoscopy on 2020/02/20

主訴

- bloody stool was noted on 2020/01/13

病史

- This 38 year old female denied any systmic diseases.
  According to her statement, bloody stool(dark red) was incidentally found on 2020/01/13, she went to LMD for help. At LMD, sigmoidoscopy was done and rectal tumor was found. She then went to our OPD in the next day for second oponion. After discussion with doctor, she was admitted on 2020/01/16 for scheduled per-anal excision of tumor and pathology showed adenocarcinoma, moderately differentiated. Other symptoms such as weight loss, fever, altered bowel habits, diarrhea, constipation, abdominal pain was all denied.
  This time, she was admitted for scheduled low anterior resection with colo-anal anastomois laparoscopy.

體檢發現

- Anal inspection:
  Recto digital examination:
  Sigmoidoscopy:
  Anoscopy:
  Abdomen:

  20200213 Sedative colonoscopy at 3F OR was done upto cecum. Excellent preparation, normal mucosa. Granulation tissue and stiches noted over previous per-anal excision site. Hemorrhoid (+)
  - General appearance :
    - easy looking , conscious clear , without cardiopulmonary distress
    - body weight : 46 kg
    - Vital signs自動帶入date 體溫(BT) 脈搏(P) 呼吸(R) 血壓(BP) 身高(BH) 體重(BW) BMI
      20200219 36.2℃ 78次/分 18次/分 107/66mmHg 157cm 46.6kg 18.9
    - Vital signs: B.P. 107/66 mmHg , P.R. 78 /min , R.R. 18 /min , B.T. 36.2 ℃
  - HEENT :
    - Eyes: no anemic conjunctiva, no icteric sclera, normal EOM
    - ENT: no otorrhea, no rhinorrhea, no erythematous mucosa, no injected tonsil, no deformity
  - Neck: supple, freely movable, trachea midline,no deformity, no engorged jugular vein , no palpable thyroid or lymph node enlargement
  - Chest:
    - Inspection: Grossly normal, symmetric and fully expansion, no accessory muscle used, no operation scar, no spider angiomata, no gynecomastia
    - Palpation: no palpable mass or nodule, no subcutaneous emphysema, no tenderness
    - Ausculation: clear breathing sound
  - Heart:
    - Palpation: no thrill, no heave
    - Percussion: normal heart size
    - Ausculation: regular heart beat, normal S1, S2, no gallop, no murmur
  - Extremities: No deformity, full ranged of motion without limitation, no clubbing finger or nail cyanosis, no pitting edema, no ulcer or pigmentation, no obvious skin lesion, no varicose vein, no bone, joint or muscle tenderness

  CRS CONDITION
  - Karnofsky performance status: (100) Normal, no complaints, no evidence of disease
  - PE of Abdomen:
  - CEA: 1.24 ng/ml
  - Imaging Studies:
  - Colonoscope report: -
  - Figures:
    1. colon & rectum

手術日期及方法

- 手術日期及方法20200220 Low anterior resection with colo-anal anastomosis Laparoscopy

住院治療經過

- This week, the patient recieved Lapa LAR and tolerated it fairly. The patient recovered smoothly. under relative stable condition, the paitent was discharged on 2020/02/26 and arranged OPD follow up.

合併症

- nil

一般檢查

- 累積報告 :

DATE	TIME	ALB	TP	BIL.T	BIL.D	ALKP	AST	ALT	LDH	CHOL	TG	HDL-C	LDL	UA	NH3	AMY
1090220	1608			0.60	0.15	68	21	10								
1090224	0856	3.1		0.43	0.15		12	5								

DATE	TIME	WBC	RBC	HGB	HCT	MCV	PLT	BLST	NEUT	BAND	SEG	LYM	MON	EOS	BAS
1090220	1611	11350		13.8	39.8		163		86.7			10.5	2.6	0.1	0.1
1090224	0924	5780	3.66	11.5	33.6	91.8	207		67.7			23.5	5.9	2.6	0.3

DATE	TIME	NA	K	CL	CA	BUN	CREAT	Mg	P	CRP	ESR	PCT	LIPASE	LACTATE
1090220	1608	137	4.4		8.0	8	0.61							
1090224	0839													6.9
1090224	0856	142	3.4	109	7.6	5	0.55	1.6	2.4					

- 放射線報告 :檢驗項目 **:CT ABDOMEN-SURVEY** 報告日期 **:1090214**
  主 訴：
  Result:
  Finding:
  1. Imaging Modality and protocol:
  CT of abdomen with IV and oral contrast.
  2. Tumor location and size:
  Rectal Ca, s/p polypectomy; Mild circumferential wall thickening of rectum; the tumor location and size are difficult to be determined.
  3. Tumor invasion:

- Dirty perirectal fat planes.
 4. Regional lymph node metastasis:
   Not assessable.
 5. Distant metastasis:
   Not assessable
 6. Other findings:
 > Presence of a tiny low density mass in left lobe of liver(S3), in favor of hepatic cyst.
 > the appearance of spleen, biliary system, pancreas and both kidneys is not unusual
 > Presence of cystic soft tissue mass arising from adnexa, right 19 mm and left 22 mm in diameter, in favor of ovary; intrauterine mucosal enhancement and fluid accumulation; fluid accumulation within cul-de-sac; please GYN clinical correlation.
 Impression:
 > rectal Ca, for staging.

檢驗項目 **:CHEST PA VIEW** 報告日期 **:1090219**
[illegible] 訴：
Result:
 Finding:
 > The cardiac shadow is within normal limit.
 > Mild increased infiltration over bilateral lung field.
 Impression:
 As above description.

特殊檢查

病理報告

其它

出院時情況

- 3.改門診治療

出院指示

藥名	劑量	劑量單位	用法	頻次	數量	數量單位	現況	天數	單位
Gaslan tab 40mg	1	TAB	PO	QID	20	TAB	TH	5	天
Lactam tab 500mg	1	TAB	PO	QID	20	TAB	TH	5	天
(□)Takepron OD tab 30mg	1	TAB	PO	QDAC	5	TAB	TH	5	天

出院計劃

[illegible]治醫師 2062F [illegible]鋒帆 / [illegible]院醫師 2328K [illegible]俊凱
正式摘要**(電子簽章：000000186432682)**

W83 / W83

doctorRank:B
